# Cytomegalovirus Infection-Associated Hemophagocytic Lymphohistiocytosis and Histiocytic Necrotising Lymphadenitis Progressing to Systemic Lupus Erythematosus: A Case Report

**DOI:** 10.7759/cureus.100871

**Published:** 2026-01-05

**Authors:** Madhushan Ranabahu, Sachinthana Sumanasekara, Dilini Wickramaratne, Vasana Mendis, Prasanna Weerawansa

**Affiliations:** 1 Department of Medicine, Postgraduate Institute of Medicine, University of Colombo, Colombo, LKA; 2 Department of Pathology, University of Peradeniya, Kandy, LKA; 3 Department of Pathology, Base Hospital - Thambuththegama, Anuradhapura, LKA; 4 Department of Pathology, Rajarata University of Sri Lanka, Anuradhapura, LKA; 5 Department of Medicine, Rajarata University of Sri Lanka, Anuradhapura, LKA

**Keywords:** cytomegalovirus, hemophagocytic lymphohistiocytosis, histiocytic necrotising lymphadenitis, lupus nephritis, systemic lupus erythematosus

## Abstract

Systemic lupus erythematosus (SLE) is an autoimmune multisystem disorder predominantly seen in young females. Its clinical presentation is enormously heterogeneous. Genetic, epigenetic and environmental factors cause SLE. Secondary hemophagocytic lymphohistiocytosis (HLH) associated with cytomegalovirus (CMV) infection is observed in immunocompromised patients and is rare in immunocompetent patients. Histiocytic necrotising lymphadenitis (HNL) is a self-limiting hyperimmune reaction rarely associated with HLH. CMV infection can be associated with HLH, HNL, and SLE. In this case, we discuss an immunocompetent young female patient who presented with an upper respiratory tract infection, which led to HLH and HNL associated with CMV infection. She recovered with initial intravenous methylprednisolone pulse immunosuppressive treatment. However, the patient later evolved into SLE.

## Introduction

Hemophagocytic lymphohistiocytosis (HLH) is a haematological emergency with a high mortality rate in the absence of proper intervention. The incidence of HLH is about 1.2 per million patients per year, with a 47% mortality rate [1]. Causes for HLH can be primary or secondary. Primary HLH typically occurs in the first few years of life due to genetic mutations in immune down-regulatory proteins and impaired natural killer cell activity [1]. Secondary HLH occurs due to various triggering factors such as infections, systemic autoimmune diseases, malignancy, pregnancy and immunosuppressant drugs [1,2]. Viral infections are frequent causes of HLH, with herpesviruses being the most common, including Epstein-Barr virus, herpes simplex virus, and cytomegalovirus (CMV) [1,2]. HLH due to CMV infection is primarily observed in immunocompromised patients, in whom reactivation of latent infection is common [1,2]. Histiocytic necrotising lymphadenitis (HNL) is a hyper-immune reaction mediated by T lymphocytes and histiocytes [3]. HNL is commonly recognised in young females and is known as Kikuchi-Fujimoto disease. Identified triggers for HNL include infections such as herpesviruses and parvovirus B19; systemic autoimmune diseases such as Still's disease, systemic lupus erythematosus (SLE), mixed connective tissue disease, and antiphospholipid syndrome; vaccination; and, rarely, HLH [3]. Clinical manifestations of HNL are fever, painful cervical lymphadenopathy, splenomegaly, leukopenia and rash [3]. HLH, HNL, and SLE demonstrate significant immunological and clinical overlap and are increasingly recognised to be associated with CMV infection [3].

## Case presentation

**Figure 1:**
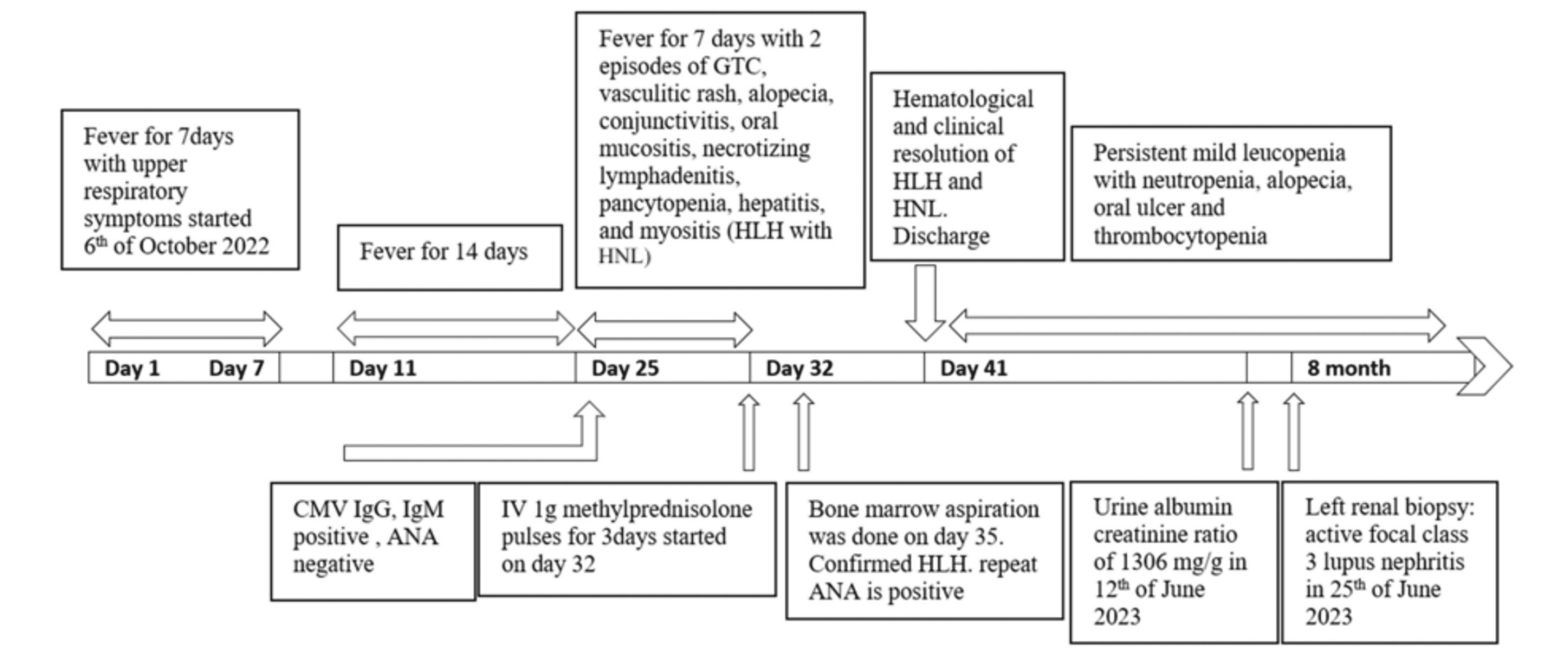
Timeline illustrating the chronological progression of the patient’s illness over time. GTC: generalised tonic-clonic seizures; HLH: hemophagocytic lymphohistiocytosis; HNL: histiocytic necrotising lymphadenitis; CMV: cytomegalovirus; ANA: antinuclear antibody

A 21-year-old previously healthy female presented with a prolonged febrile illness. Her fever began on 6 October 2022 and was intermittent, accompanied by chills, malaise, arthralgia, and a dry cough without shortness of breath. The initial febrile episode lasted for seven days and then resolved. She remained afebrile for the following three days. Subsequently, the fever recurred intermittently, with arthralgia, myalgia, and loss of appetite, and persisted for approximately two weeks (Figure 1).

**Figure 2:**
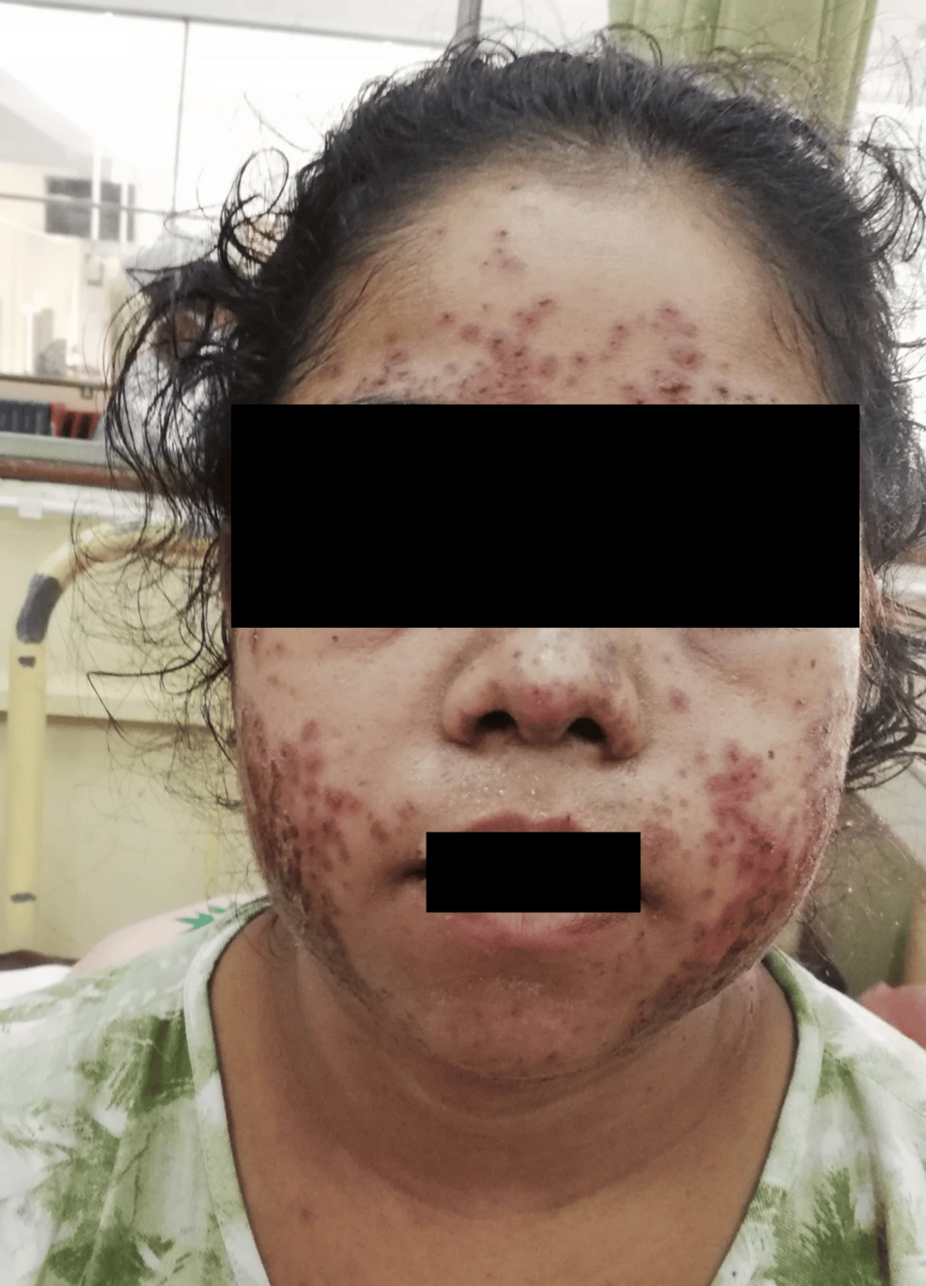
The image of the face and upper chest shows diffuse erythematous-violaceous papules and plaques distributed across the forehead, periorbital region, cheeks, and upper chest. Many lesions show central petechiae. There is marked bilateral periorbital and generalised facial swelling with mild diffuse hair loss over the frontal hairline. The patient provided written and signed consent for publication of this identifiable facial image in an open-access journal.

The fever continued and was accompanied by the gradual appearance of a painless, non-pruritic rash that began on the face and ears and extended to the upper chest. The patient also had red eyes, bleeding mouth and excessive hair loss during this period. The patient was admitted to a local hospital on day 25 of illness. The examination showed a vasculitic rash over her face, sparing nasolabial folds and involving her ears and upper chest. The patient had painless bilateral conjunctivitis, subconjunctival haemorrhages with periorbital swelling, painless mucositis, and non-scarring alopecia (Figure 2).

**Figure 3:**
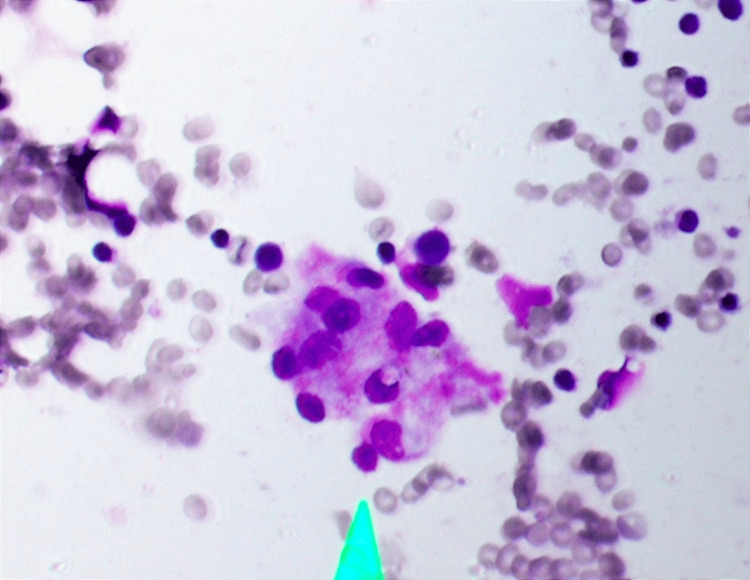
Hematoxylin and eosin-stained bone marrow aspirate specimen showing hemophagocytosis (light green arrow).

Tender, discrete, bilateral cervical lymphadenopathy was present in both the anterior and posterior groups. Blood pressure was 110/70 mmHg with a regular heart rate of 90 bpm. Abdominal examination revealed no organomegaly. The rest of the examination, including the funduscopic examination, was unremarkable. At the local hospital, her clinical condition deteriorated, and the patient developed two episodes of generalised tonic-clonic convulsions, each lasting for less than one minute, pancytopenia, hepatitis, and myositis. A clinical diagnosis of a severe form of SLE was suspected, and the patient was started on intravenous methylprednisolone 1g daily pulse on day 32 of illness at the local hospital. However, the initial antinuclear antibody (ANA) test was negative. The patient was transferred to the Teaching Hospital, Anuradhapura, on day 32 of illness for further evaluation and treatment. Intravenous methylprednisolone 1g daily for a total of three days was continued. At this point, a clinical diagnosis of secondary HLH was made based on clinical and investigative findings. Non-contrast CT of the brain was normal. A bone marrow biopsy was performed on day 35, which revealed hemophagocytosis (Figure 3).

**Figure 4:**
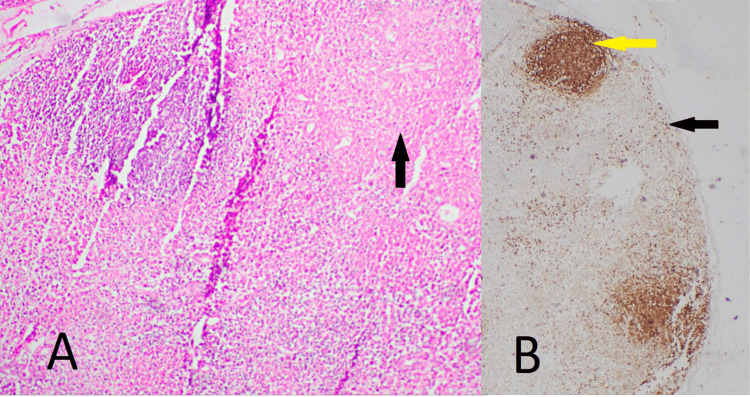
A: Cervical lymph node with evidence of necrotising lymphadenitis. The black arrow shows a necrotising area (hematoxylin and eosin, X100). B: Immunohistochemistry of the cervical lymph node. The black arrow indicates CD3-positive T lymphocytes in paracortical areas and around foci of necrosis. The yellow arrow shows CD20-positive B lymphocytes in residual lymphoid follicles (X40).

The patient's initial investigations were positive for CMV IgG and IgM antibodies, as reported by the local hospital. The cervical lymph node biopsy revealed necrotising lymphadenitis (Figure 4).

**Figure 5:**
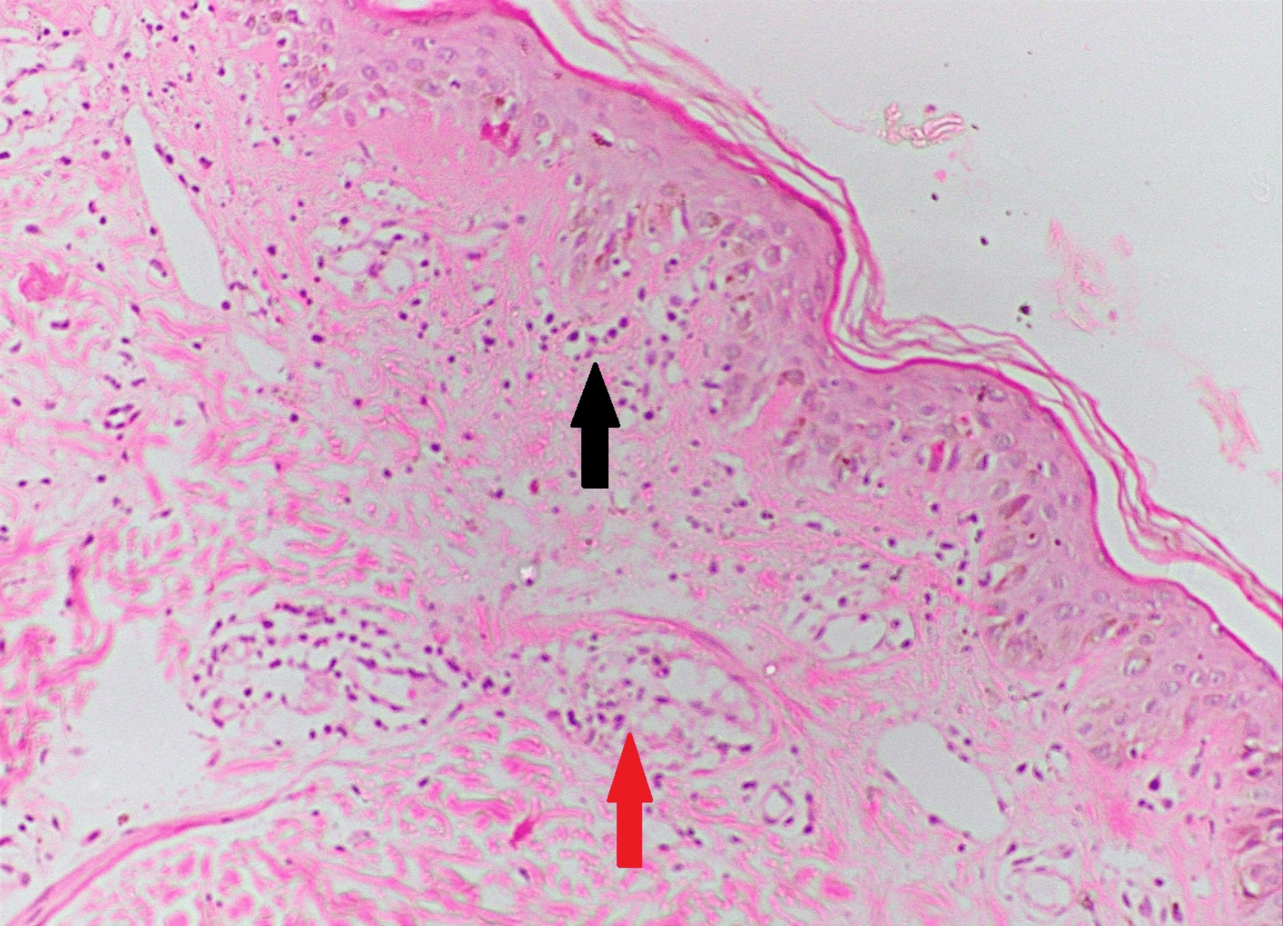
Histology of the skin vasculitic rash. The black arrow indicates interface dermatitis, and the red arrow shows lymphocytic vasculitis (hematoxylin and eosin, ×200).

**Figure 6:**
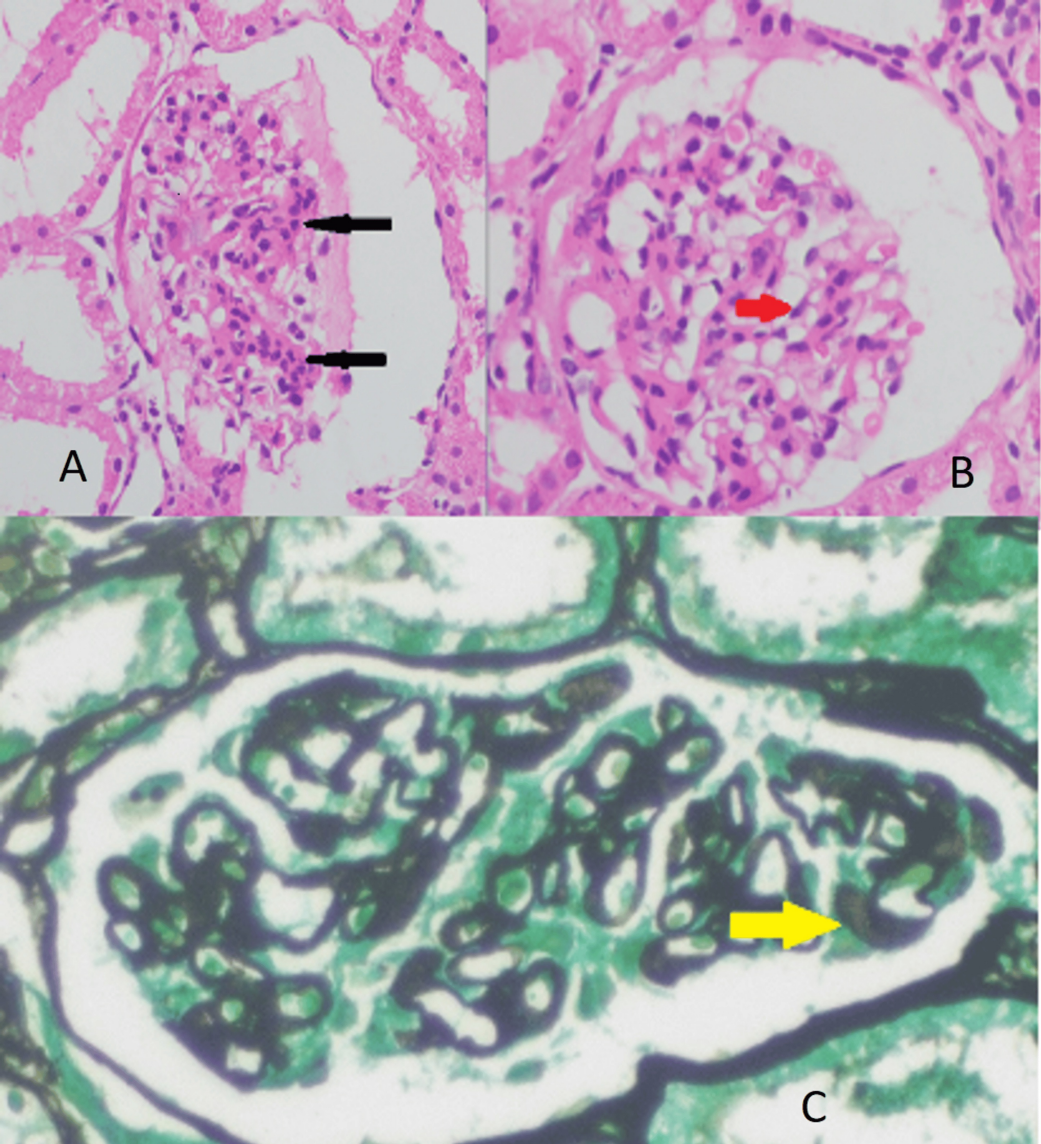
(A, B) Hematoxylin and eosin-stained sections of the renal biopsy. The black arrows indicate subtle endocapillary proliferation and karyorrhexis, and the red arrow indicates mild mesangial proliferation with matrix expansion. Wire-loop lesions, hyaline deposits, and crescents are absent. There is no interstitial inflammation, tubular atrophy, or interstitial fibrosis. (C) Methenamine silver-stained section showing a glomerulus with focal, subtle spike formation in the glomerular basement membrane (yellow arrow).

The skin biopsy of the lesion showed interface dermatitis with lymphocytic vasculitis (Figure 5). The diagnosis of HLH with HNL associated with CMV infection was made retrospectively (Table 1). Repeated ANA was positive on day 35 of illness. The patient clinically improved with treatment. Her skin lesions, cervical lymphadenitis, pancytopenia, and general well-being improved. She didn’t develop further neurological manifestations. CMV PCR was performed on day 40 of illness and was negative. The patient was discharged on day 41. Alopecia, oral ulcers, mild lymphopenia, neutropenia and thrombocytopenia persisted throughout her outpatient follow-up. The patient was started on oral cyclosporine 25mg once a day with hydroxychloroquine 200mg once a day for her symptoms. Later, the patient was found to have persistent clinical albuminuria. Her urine albumin to creatinine ratio done on 12 June 2023 was 1306mg/g, and a renal biopsy was required. The renal biopsy showed active focal class 3 and impending class 5 lupus nephritis on 25 June 2023 (Figure 6). The immunofluorescence studies showed 3+ granular positivity for IgG and 2+ granular positivity for IgA, IgM, and C3 in both the mesangium and the capillary walls, indicating a full-house pattern. The above features are suggestive of active class 3 lupus nephritis with impending class 5 nephritis, with an activity score of 4/24 and a chronicity score of 0/12.

**Table 1:** Laboratory investigations. ANC: absolute neutrophil count; anti-Sm: anti-Smith antibody; APTT: activated partial thromboplastin time; C3: complement 3; C4: complement 4; dsDNA: double-stranded DNA antibody; ESR: erythrocyte sedimentation rate; HIV: human immunodeficiency virus

Investigation (Reference Range)	Day 25	Day 32	Day 38
White blood cell count (4-10x10^3^/µL)	20.3	1.13	3.81
ANC (1500-8000/µL)	1112	720	1710
Haemoglobin (11-16g/dL)	11.6	9.0	9.3
Platelets (150-400x10^3^/µL)	140	53	113
C-reactive protein (mg/dL)	3.5	7.3	-
Procalcitonin (ng/mL)	-	0.33	-
ESR (mm/1^st ^hour)	40	50	-
Serum creatinine (µmol/L)	88.4	50	-
Urine albumin creatinine ratio (mg/g)	52.7	-	-
Antinuclear antibody	Negative	1:1280	-
Anti-Sm antibody	Negative	-	-
dsDNA antibody	Negative	-	-
Rheumatoid factor (<8IU/L)	<8	-	-
Anti-Jo 1 antibody	Negative	-	-
U1RNP antibody	Negative	-	-
Anti-SCL 70 antibody	Negative	-	-
Anti U1RNP antibody	Negative	-	-
Anti-La/SSB antibody	Negative	-	-
Anti-Ro/SSA antibody	Positive	-	-
Anti-beta 2 glycoprotein antibody IgG	Negative	-	-
Anti-beta 2 glycoprotein antibody IgM	Negative		
Anti-cardiolipin antibody IgG	Negative	-	-
Anti-cardiolipin antibody IgM	Negative		
C3 (80-160mg/dL)	30	-	-
C4 (16-48mg/dL)	10	-	-
Serum ferritin (24-336mg/dL)	3258	-	-
Lactate dehydrogenase level (10-280U/L)	1844	1738	796
Creatine phosphokinase (34-145U/L)	629	-	-
Cytomegalovirus virus IgM	Positive	-	-
Cytomegalovirus virus IgG	Positive		
Epstein-Barr virus IgM	Negative	-	-
Hepatitis B surface antigen	Negative	-	-
Hepatitis C antibody	Negative	-	-
Dengue virus IgM	Negative	-	-
HIV 1&2 (antigen/antibody)	Negative	-	-
Ig A level (40-350mg/dL)	-	322	-
Ig G level (650-1600mg/dL)	-	1249	-
IgA level (0-300mg/dL)	-	222	-
Serum protein electrophoresis	No M bands	-	-
Aspartate transaminase (10-35U/L)	-	410	79
Alanine transaminase (13-40U/L)	-	110	68
Gamma-glutamyl transferase (7-50U/L)	-	55	-
Alkaline phosphatase (30-120U/L)	137	-	-
Prothrombin time (PT)	-	12 seconds	-
APTT	-	36 seconds	-
Albumin (35-52g/l)	3.94	-	-
Globulin (20-35g/l)	3.37	-	-
Total bilirubin (5-21µmol/L)	0.68	-	-
Direct bilirubin (1.7-5.1µmol/L)	0.29	-	-
Urine full report	Albumin nil, pus cells 2-3, red cells 3-4	-	-
Triglyceride (<150mg/dL)	-	235	-
Blood culture	Negative	Negative	-
Urine culture	Negative	Negative	-

The patient was started on mycophenolate mofetil at a dose of 1g twice daily along with oral prednisolone 10mg once daily. She showed both clinical and biochemical improvement with immunosuppressive therapy.

## Discussion

Prolonged fever associated with multi-organ injury is a clinical feature of HLH, which is also seen in sepsis, Still’s disease, catastrophic anti-phospholipid syndrome, acute liver failure, thrombotic thrombocytopenic purpura and hemolytic uremic syndrome. The pathophysiology of most of these conditions involves tissue hypoperfusion, leading to hypoxic cellular injury. In HLH, tissue injury results from infiltration by macrophages, cytotoxic T cells, and natural killer cells, with intact tissue perfusion. The 2004 HLH diagnostic criteria are used to diagnose HLH [1,2,4]. An acute febrile illness with respiratory symptoms is a common presentation of acute CMV infection in immunocompetent patients [5]. CMV infection can be diagnosed using blood samples for CMV IgG, IgM and PCR. Both CMV IgG and IgM are positive in recent primary CMV infection and in CMV reactivation [6]. Hence, it is difficult to differentiate primary CMV infection from reactivation. CMV IgG avidity testing is beneficial, showing low avidity for primary CMV infection and high avidity for reactivation [6]. The patient’s CMV IgG and IgM were positive during the initial febrile phase, supporting the diagnosis of CMV infection. However, the CMV PCR was performed after recovery from HLH syndrome, and it was negative. A negative CMV PCR result may reflect clearance of CMV viremia and seroconversion in this case. The diagnosis of CMV infection in this case was primarily based on CMV serological studies. SLE patients typically have positive CMV IgG and IgM antibodies, primarily due to reactivation of latent CMV infection during immunomodulator therapy [7]. There was no family history of immunodeficiency diseases. Screening tests for diabetes mellitus, retroviral infection, and immunoglobulin levels were normal. The patient met four of the 2004 HLH diagnostic criteria: prolonged fever, pancytopenia, bone marrow hemophagocytosis, and elevated serum ferritin. Cytopenia is a recognised feature of HLH, attributable to excessive, dysregulated macrophage-mediated engulfment of bone marrow cells [1,2,4].

The patient’s bone marrow aspiration showed hemophagocytosis (Figure 3). Generalised tonic-clonic convulsion is most likely a manifestation of HLH, as neurological manifestations are seen in 46% of patients as generalised seizure, nerve palsies, coma, and opisthotonus [8]. Dermatological manifestations of HLH are maculopapular, erythrodermic, purpuric, petechiae, and morbilliform eruptions [9]. Figures 2-5 show the patient's skin lesions and their histology, respectively. Skin biopsy rarely shows hemophagocytosis. Interface dermatitis and lymphocytic vasculitis are common histological findings in skin biopsies from patients with HLH, as observed in this patient [9]. Lymphadenopathy in HLH is a recognised feature, and biopsy shows classically hemophagocytosis [1,2,4]. However, the patient’s cervical lymph node biopsy showed necrotising lymphadenitis (Figure 4). Necrotising lymphadenitis can be seen in HNL (also known as Kikuchi-Fujimoto disease) or SLE.

Chen et al. have described five CMV-negative adult patients with HLH with HNL. Two patients developed SLE during follow-up, as seen in this patient [10]. HLH requires prompt treatment to avoid irreversible multi-organ failure and death. However, establishing the diagnosis of HLH is essential. The principles of HLH management include treating the trigger (familial or acquired) and managing hyperimmune cytokine storms with immunosuppressants [4,10]. Steroids or anti-cytokine agents, such as anakinra and tocilizumab, are recommended for the treatment of virus-induced HLH [11]. In this case, HLH was successfully treated with IV methylprednisolone. The patient was diagnosed with SLE lupus nephritis according to the 2019 European League Against Rheumatism/American College of Rheumatology criteria after eight months following CMV infection-associated HLH with HNL. Current evidence indicates a probable association between viral infections and autoimmune diseases. CMV viral antigens interact with host innate and adaptive immune systems, leading to molecular mimicry and autoimmunity, which remains hypothesised [7,12]. Six cases in the literature described CMV infection in immunocompetent patients complicated with HLH (Table 2). This case was the only one in which CMV infection was associated with HLH and HNL, and it later progressed to SLE during follow-up.

**Table 2:** Literature review. ANA: antinuclear antibody; CMV: cytomegalovirus; dsDNA Ab: double-stranded DNA antibody; IgG: immunoglobulin G; IgM: immunoglobulin M; PCR: polymerase chain reaction; SLE: systemic lupus erythematosus

Author	Age and Sex	Initial Clinical Features	CMV Serology	Autoimmune Antibody for SLE	Follow-Up
Tsuda and Shirono, 1996 [13]	21-year-old female	Fever, cervical lymphadenopathy	IgG (+) IgM (+)	ANA negative	No SLE features for six months
Hot et al., 2008 [14]	32-year-old female	Fever, chills, malaise, cough, night sweats	IgG (+) low avidity IgM (+) PCR (+)	ANA negative, dsDNA Ab negative	No SLE features for 24 months
Atim-Oluk, 2013 [15]	48-year-old female	Lethargy, dyspnea, right hypochondrial pain	IgG (+) low avidity IgM (+) PCR (+)	ANA negative, dsDNA Ab negative	No SLE features for six months
Bonnecaze et al., 2017 [1]	39-year-old female	Nausea, vomiting, diarrhoea, abdominal pain, fever, night sweats	IgG (+) IgM (+) PCR (+)	ANA negative, dsDNA Ab negative	No SLE features for two months
Singh et al., 2020 [16]	18-year-old male	Fever, dry cough	PCR (+)	ANA negative	Died during an acute illness
Elliott et al., 2022 [17]	34-year-old male	Fever, sore throat, cough, malaise, headache	IgG (+) low avidity IgM (+) PCR (+)	ANA negative	No SLE features for 10 months

## Conclusions

This is a case of a 21-year-old immunocompetent healthy female patient who presented with prolonged febrile illness and was found to have a CMV infection associated with HLH and HNL. The patient completely recovered from HLH and HNL with intravenous methylprednisolone pulse treatment. Later, the patient was diagnosed with SLE lupus nephritis during follow-up. Hence, CMV infection can be associated with HLH, HNL and later autoimmunity such as SLE. This case study shows the importance of long-term autoimmunity monitoring following the CMV infection-associated HLH or HNL cases.
